# International Spread of Tet(X4)-Producing *Escherichia coli* Isolates

**DOI:** 10.3390/foods11142010

**Published:** 2022-07-07

**Authors:** Zengfeng Zhang, Zeqiang Zhan, Chunlei Shi

**Affiliations:** MOST-USDA Joint Research Center for Food Safety, School of Agriculture and Biology, and State Key Laboratory of Microbial Metabolism, Shanghai Jiao Tong University, Shanghai 200240, China; zhanzengfeng118@163.com (Z.Z.); zhanzeqiang@sjtu.edu.cn (Z.Z.)

**Keywords:** *E. coli*, *tet*(X4), phylogenomic analysis, IS*CR2*

## Abstract

Tigecycline resistance in bacteria has become a significant threat to food safety and public health, where the development of which is attributed to plasmid-mediated *tet*(X4) genes. In this study, the genomes of 613 *tet*(X4)-producing *Escherichia coli* (*E. coli*) isolates, available from public databases, are evaluated to determine their international prevalence and molecular characterization. These *E. coli* isolates have been disseminated in 12 countries across Asia and Europe. It was found that pigs and their products (*n* = 162) were the most common vehicle, followed by humans (*n* = 122), chickens (*n* = 60), and the environment (*n* = 49). Carbapenems-resistant genes *bla*_NDM-5_ (1.3%) and *bla*_NDM-1_ (0.2%) were identified, as well as colistin-resistant genes *mcr*-1.1 (12.6%) and *mcr*-3.1 (0.5%). It was noted that the tigecycline-resistant gene cluster *tmex*C-*tmex*D-*topr*J1 was identified in seven (1.1%) isolates. Phylogenomic results indicated that *tet*(X4)-producing *E. coli* isolates fell into seven lineages (lineages I, II, III, IV, V, VI, and VII), and international spread mainly occurred in Asian countries, especially China, Pakistan, Singapore, and Malaysia. Four forms of *tet*(X4) transposon units were found, including the I-type (IS*26-tet*(X4)-IS*CR2*), II-type (ΔIS*1R*-*tet*(X4)-IS*CR2*), III-type (IS*CR2-tet*(X4)-IS*CR2*), and IV-type (IS*CR2-tet*(X4)-ΔIS*CR2*). These findings underline further challenges for the spread of *E. coli* bearing *tet*(X4) gene.

## 1. Introduction

Tigecycline is considered to be a drug of last resort for the treatment of multidrug-resistant (MDR) and even extensively drug-resistant bacteria. Tigecycline resistance in bacteria primarily has resulted from chromosome-mediated overexpression of efflux pumps and ribosome protection [1,2]. Until 2019, novel plasmid-mediated high-level tigecycline resistance genes *tet*(X4)/*tet*(X3) were discovered in *Enterobacteriaceae* and *Acinetobacter* isolates from animals and humans in China [3]. It was of note that Tet(X4) could degrade all tetracyclines, including tigecycline and the USFDA newly approved eravacycline [4], which poses a new threat to public health.

Currently, *tet*(X4)-producing *E. coli* isolates have been disseminated in meat products such as pork and chicken, food animals, animal feces, and farm soil [3,4,5,6,7]. In a previous study, *tet*(X4)-producing *E. coli* isolates in pigs and chickens accounted for 1.3% and 0.8%, respectively, which was disseminated in five provinces in southern and eastern China including Guangdong, Fujian, Jiangsu, Jiangxi, and Guangxi [4]. However, *tet*(X4) had a relatively high prevalence (33.3–50.0%) in both pigs and chickens in some provinces of China such as Shaanxi and Ningxia [8]. These results suggested that food animals and their meat products were still an important reservoir of *tet*(X4). It was noted that *tet*(X4)-producing *E. coli* isolates also emerged in humans in recent years, where the detection rate of *tet*(X4)-producing *E. coli* isolates from hospital patients was 4.5% [6]. The genetic link between meat/animal-borne *tet*(X4)-producing *E. coli* isolates and human-borne isolates requires urgent investigation.

Furthermore, *tet*(X4)-positive plasmids are highly transferable in *E. coli* [4], which could facilitate their dissemination among *Enterobacteriaceae* bacteria. Furthermore, the *tet*(X4) gene has also been found in existence with the colistin-resistant gene *mcr*-1 and/or the carbapenem-resistant gene *bla*_NDM-1_ [3,4,9], which has resulted in concurrent resistance to tigecycline and colistin/carbapenem. Therefore, the emergence of the plasmid-mediated *tet*(X4) gene poses a significant threat to public health which requires urgent surveillance in terms of its prevalence.

Here, we characterize an international distribution of *tet*(X4)-producing *E. coli* isolates using a data set of 613 genomes from the public database. Antimicrobial resistance (AMR) genotypes, virulence genotypes, plasmid replicon types, and phylogenomic characteristics are further analyzed here, as well as the genetic environment of *tet*(X4).

## 2. Materials and Methods

### 2.1. E. coli Genomes Collected

We searched two important words, “*tet*(X4)” and “*E. coli*”, in the public NCBI database on 2 April 2022. A total of 613 *E. coli* isolates were positive for the *tet*(X4) gene, and the detailed genome information including sample sources, countries, and years was downloaded. A total of 585 genomes were available for phylogenomic analysis because the rest had not been released.

### 2.2. AMR Genotypes, Virulence Genotypes, and Plasmid Replicon Types Identification

ResFinder 4.1 was used to identify antimicrobial resistance genes (ARGs) and chromosomal mutations mediating antibiotic resistance in the genome [10]. Virulence factors were identified using VFanalyzer (http://www.mgc.ac.cn/cgi-bin/VFs/v5/main.cgi?func=VFanalyzer (accessed on 2 June 2022) in the virulence factor database (VFDB) [11]. Plasmidfinder was used to identify replicon types of plasmids [12]. ISfinder (https://www-is.biotoul.fr/ (accessed on 2 June 2022) was used to analyze the IS and transposons in the genome.

### 2.3. Phylogenomic Analysis

A total of 585 *tet*(X4)-producing *E. coli* genomes were used for phylogenomic analysis. Single-nucleotide polymorphisms (SNPs) were extracted using Snippy (https://github.com/tseemann/snippy (accessed on 2 June 2022) to generate the core genomic alignment. Gubbins [13] was then used to identify and remove recombination regions using an algorithm that iteratively identifies loci containing elevated densities of base substitutions, then resulting in pairwise SNP differences that could be calculated. The core SNP alignment was used to generate a maximum-likelihood phylogeny using RAxML v8.1.23 [14] with the GTR nucleotide substitution model. The display, annotation, and management of phylogenetic trees were performed by the ITOL tool [15].

### 2.4. Local Database Construction and BLAST+ Comparison

In this study, 585 genome sequences were used as a local BLAST database to search the arrangement forms of *tet*(X4). First, BLAST+ was downloaded from NCBI (https://ftp.ncbi.nlm.nih.gov/blast/executables/blast+/LATEST/ (accessed on 2 June 2022), and then a database folder (585 × 4 db) was created and added as a path for the environment variant. Custom reference genome files were created using makeblastdb. The gene sequence of *tet*(X4) was used as the query file, and then the instruction “blastn-query *tet*(X4).fasta-db585X4db-out *tet*(X4)out-outfmt6” was input to generate the alignment sequence (.txt).

### 2.5. Data Analysis

The bar charts of the AMR genotypes, virulence genotypes, and plasmid replicon types were generated with GraphPad Prism 7.0 (GraphPad Software, San Diego, CA, USA). The world map marked with the distribution of *tet*(X4)-producing *E. coli* was drawn by Inkscape 0.92 (Inkscape Software, Brooklyn, NY, USA).

## 3. Results

### 3.1. Dissemination of tet(X4)-Producing E. coli in Asia and Europe

**Figure 1:**
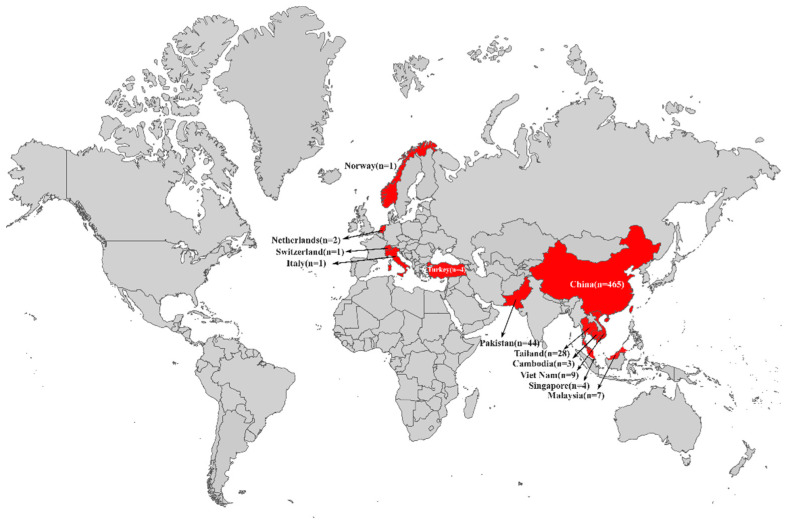
Global distribution of *tet*(X4)-producing *E. coli* isolates.

Currently, a total of 613 *tet*(X4)-producing *E. coli* genomes are available in the public databases (2 April 2022). As shown in Figure 1, these *tet*(X4)-producing *E. coli* isolates had emerged in 12 countries across Asia and Europe, including China (*n* = 465), Pakistan (*n* = 44), Thailand (*n* = 28), Vietnam (*n* = 9), Malaysia (*n* = 7), Turkey (*n* = 4), Singapore (*n* = 4), Cambodia (*n* = 3), Netherlands (*n* = 2), Switzerland (*n* = 1), Norway (*n* = 1), and Italy (*n* = 1).

### 3.2. Pig and Its Products Were the Main Vehicles of tet(X4)-Producing E. coli

**Figure 2:**
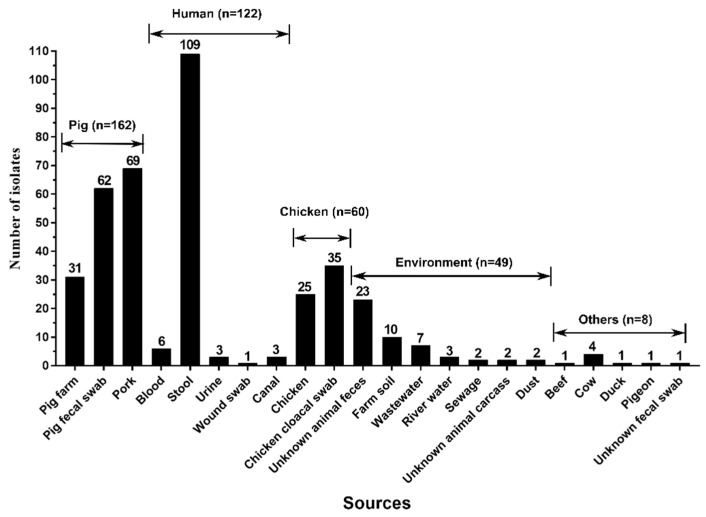
The sample sources of *tet*(X4)-producing *E. coli* isolates.

It was found that pigs and their products (*n* = 162) represented the most common source of *tet*(X4)-producing *E. coli* isolates, followed by human (*n* = 122), chicken (*n* = 60), environmental (*n* = 49), and other sources (*n* = 8) (Figure 2). Among 162 pigs and their products isolates, pork (*n* = 69) was the most common one (Figure 2). Among 122 human isolates, stool isolates (*n* = 69) were most common, followed by blood (*n* = 6), urine (*n* = 3), canal (*n* = 3), and wound swab isolates (*n* = 1). Among the 49 environment isolates, animal feces (*n* = 69) were most common, followed by farm soil (*n* = 10), wastewater (*n* = 7), river water (*n* = 3), sewage (*n* = 2), animal carcass (*n* = 2) and dust isolates (*n* = 1) (Figure 2).

### 3.3. IncHI1A and IncHI1B Were the Dominant Plasmid Types in tet(X4)-Producing E. coli

**Figure 3:**
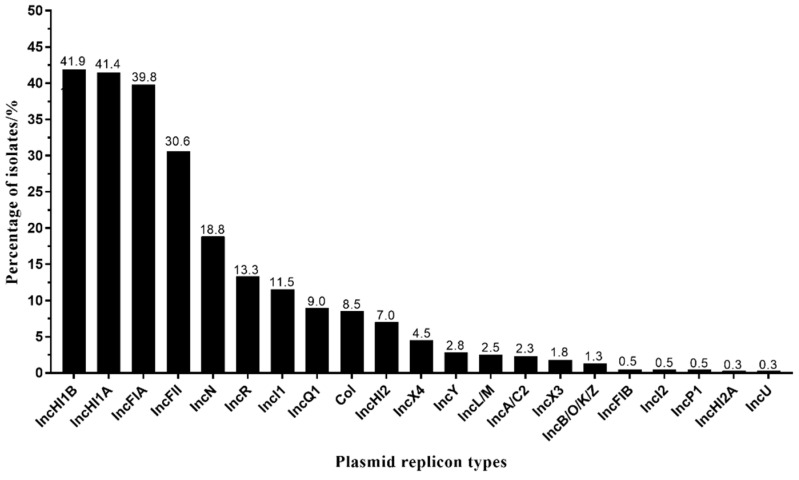
The plasmid replicon types in *tet*(X4)-producing *E. coli* isolates.

In this study, 399 *tet*(X4)-producing *E. coli* isolates were found to carry plasmid replicon genes. A total of 21 plasmid replicon types were identified (Figure 3). The most common types were IncHI1A and IncHI1B, accounting for 41.4% and 41.9%, respectively (Figure 3). The other identified plasmid types were the following: IncFIA (39.8%), IncFII (30.6%), IncN (18.8%), IncR (13.3%), IncI1 (11.5%), IncQ1 (9.0%), Col (8.5%), IncHI2 (7.0%), IncX4 (4.5%), IncY (2.8%), IncL/M (2.5%), IncA/C2 (2.3%), IncX3 (1.8%), IncB/O/K/Z (1.3%), IncFIB (0.5%), IncI2 (0.5%), IncP1 (0.5%), IncHI2A (0.3%), and IncU (0.3%) (Figure 3). In addition, 191 (27.3%) isolates were found to carry at least three plasmid replicon genes, and 63 (15.8%) isolates carried at least four replicon genes. Furthermore, 5 (1.3%) isolates carried at least six replicon genes.

### 3.4. Antimicrobial Resistance (AMR) Genotypes and Virulence Genotypes in tet(X4)-Producing E. coli

**Figure 4:**
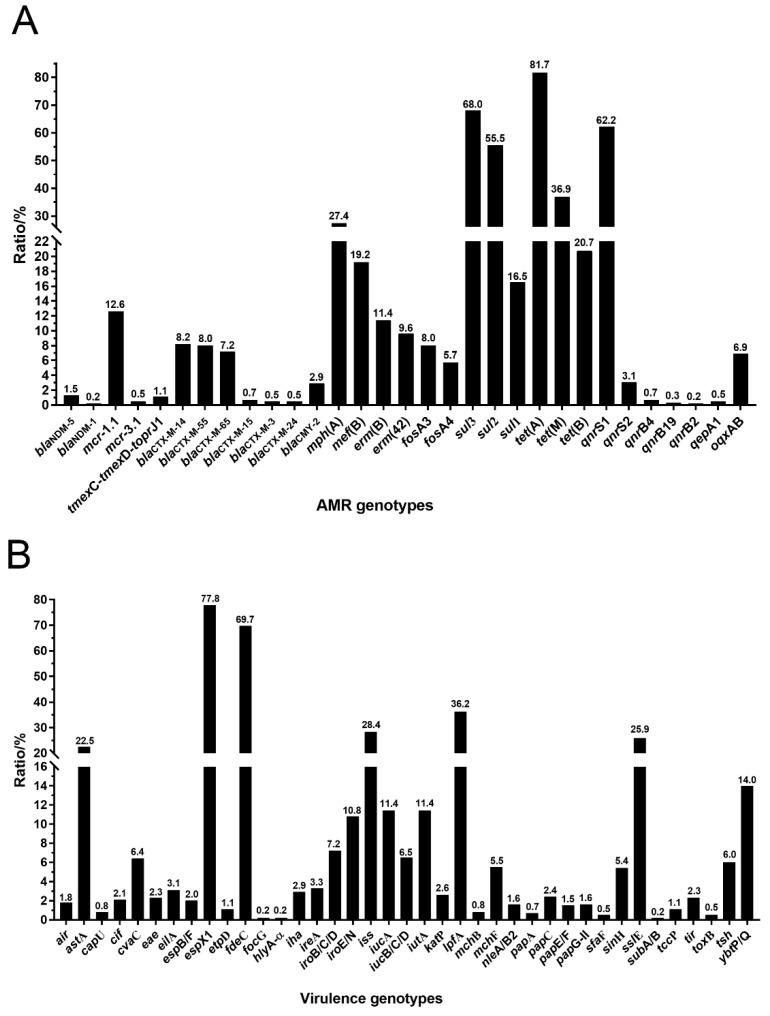
AMR genotypes (**A**) and virulence genotypes (**B**) in *tet*(X4)-producing *E. coli* isolates.

A total of 64 acquired AMR genes were found in these *tet*(X4)-producing *E. coli* isolates (Figure 4A and Table S1). Tetracycline-resistant genes *tet*(A) (81.7%), *tet*(M) (36.9%) and *tet*(B) (20.7%) were identified. Furthermore, carbapenems-resistant genes *bla*_NDM-5_ (1.3%) and *bla*_NDM-1_ (0.2%) were identified, as well as colistin-resistant genes *mcr*-1.1 (12.6%) and *mcr*-3.1 (0.5%). It was interesting that a tigecycline-resistant gene cluster *tmex*C-*tmex*D-*topr*J1 was identified in seven (1.1%) isolates. In addition, a total of six *bla*_CTX-M_ variants were identified, where *bla*_CTX-M-14_ (8.2%) was the most common one, followed by *bla*_CTX-M-55_ (8.0%), *bla*_CTX-M-65_ (7.2%), *bla*_CTX-M-15_ (0.7%), *bla*_CTX-M-3_ (0.5%), and *bla*_CTX-M-24_ (0.5%). Gene *mph*(A) (27.4%) was the most common macrolides-resistant gene, followed by *mef*(B) (19.2%), *erm*(B) (11.4%), and *erm*(42) (9.6%). Fosfomycin-resistant gene *fos*A3 and *fos*A4 accounted for 8.0% and 5.7%, respectively. Gene *qnr*S1 (62.2%) was the most common plasmid mediated quinolones-resistant (PMQR) gene, followed by *oqx*AB (6.9%), *qnr*S2 (3.1%), *qnr*B4 (0.7%), *qep*A1 (0.5%), *qnr*B19 (0.3%) and *qnr*B2 (0.2%). Mutations in genes associated with AMR from genomes were also identified. It was found that *glp*T_E448K (57.3%) was the most common mutation, followed by in *gyr*A_S83L (39.5%), *par*C_S80I (26.6%), *gyr*A_D87N (22.7%), *uhp*T_E350Q (7.5%), *par*E_S458A (6.0%), *cya*A_S352T (5.1%), *par*C_A56T (3.3%), *nfs*A_G131D (2.3%), *sox*S_A12S (1.5%), *nfs*A_R15C (1.1%), *par*E_L416F (0.7%), *par*E_I355T (0.3%), *nfs*A_Q44STOP (0.3%), and *mar*R_S3N (0.2%) (Table S1).

A total of 48 virulence genes were identified in these *tet*(X4)-producing *E. coli* isolates (Figure 4B). Gene *esp*X1 was the most common virulence factor, accounting for 77.8%, followed by *fde*C (69.7%), *lpfA* (36.2%), *iss* (28.4%), *ssl*E (25.9%), *ast*A (22.5%), *ybt*P/Q (14.0%), *iuc*A (11.4%), *iut*A (11.4%), *iro*E/N (10.8%), *iro*B/C/D (7.2%), *iuc*B/C/D (6.5%), *cva*C (6.4%), *tsh* (6.0%), *mch*F (5.5%), *sin*H (5.4%), *ire*A (3.3%), *eil*A (3.1%), *iha* (2.9%), *kat*P (2.6%), *pap*C (2.4%), *eae* (2.3%), *tir* (2.3%), *cif* (2.1%), *esp*B/F (2.0%), *air* (1.8%), *nle*A/B2 (1.6%), *pap*G-II (1.6%), *pap*E/F (1.5%), *etp*D (1.1%), *tcc*P (1.1%), *cap*U (0.8%), *mch*B (0.8%), *pap*A (0.7%), *sfa*F (0.5%), *tox*B (0.5%), *foc*G (0.2%), *hly*A-α(0.2%), and *sub*A/B (0.2%).

### 3.5. Phylogenomic Analysis of tet(X4)-Producing E. coli

**Figure 5:**
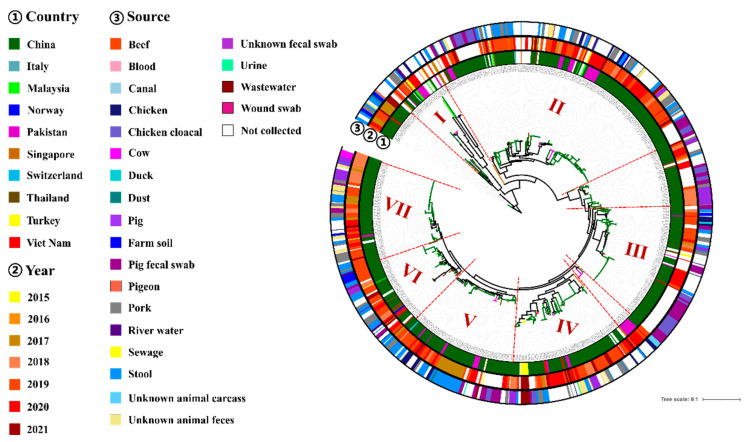
Phylogenetic tree of *tet*(X4)-producing *E. coli* genomes from different countries. Circle ① depicts isolate isolation countries; circle ② denotes the distribution of years; circle ③ denotes sample sources. The detailed information in circles ①–③ using various colors is shown in the key. Lineages I, II, III, IV, V, VI, and VII were shown in the figure.

Phylogenomic analysis was performed for 585 *tet*(X4)-producing *E. coli* genomes from 10 countries to provide their evolutional features. A total of 31,260 core SNPs were identified to construct a maximum likelihood tree (Figure 5). Phylogenomic results indicated that *tet*(X4)-producing *E. coli* isolates might have emerged early in China because they were the base of the phylogenetic tree, then evolving into lineages I, II, III, IV, V, VI, and VII (Figure 5). All these seven lineages were mixed clusters, which were composed of isolates from different countries. Lineage I was composed of isolates from China, Pakistan, Singapore, and Malaysia. Lineage II was composed of isolates from seven countries including China, Pakistan, Thailand, Singapore, Malaysia, Vietnam, and Italy. Isolates from Turkey fell into lineage IV, together with those from China, Pakistan, and Vietnam. Isolates from China clustering with those from Pakistan could be found in lineages I, II, III, IV, V, and VII. Isolates from China clustering with those from Thailand could be found in lineages II, III, V, VI, and VII. Similar results were also found in isolates from China with those from Malaysia and Vietnam, Furthermore, some isolates from European countries were also found in these lineages, such as Italy (lineage II), Norway (lineage VI), and Switzerland (lineage V), suggesting a close genetic relationship with those from Asia. These results indicated that the international spread of *tet*(X4)-producing *E. coli* isolates has occurred in Asian countries, especially China, Pakistan, Singapore, and Malaysia. It was noted that the *tet*(X4)-producing *E. coli* might have been introduced into Europe from Asia.

Clone spread of *tet*(X4)-producing *E. coli* isolates was found among different sources. For example, some isolates from stool, blood, pork, and unknown animal feces in lineage IV shared a high similar genetic type (Figure S1). Similar results were also found in some stool, blood, pig, pork, pig fecal swab, cow, and unknown animal feces isolates in lineage VII (Figure 5). It was noted that the genetic types of some chicken cloacal isolates could be highly similar to those from pig fecal swabs in lineage III, which suggested that the spread of *tet*(X4)-producing *E. coli* isolates was likely to occur between chickens and pigs.

### 3.6. Genetic Environment of tet(X4) in E. coli

**Figure 6:**
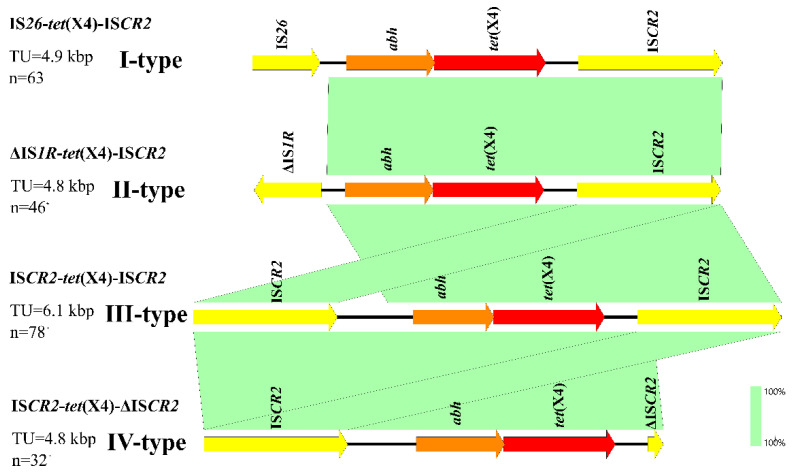
Genetic environment of *tet*(X4) in *E. coli*. Areas shaded in light green indicate homologies between the corresponding genetic loci.

In this study, four forms (I, II, III, and IV) of *tet*(X4) transposon units were identified (Figure 6). The I-type of *tet*(X4) was linked to the transposable elements IS*26* upstream and IS*CR2* downstream. The I-type pattern of IS*26-tet*(X4)-IS*CR2* was identified in 63 isolates, and its size was approximately 4.9 kb in length. The II-type of *tet*(X4) was linked to the truncated ΔIS*1R* upstream and IS*CR2* downstream. The II-type pattern of ΔIS*1R-tet*(X4)-IS*CR2* was identified in 46 isolates, and its size was approximately 4.8 kb in length. The III-type of *tet*(X4) was linked to the IS*CR2* upstream and IS*CR2* downstream. The III-type pattern of IS*CR2-tet*(X4)-IS*CR2* was the most common arrangement profile (*n* = 78), and its size was approximately 6.1 kb in length. The IV-type of *tet*(X4) was linked to the IS*CR2* upstream and truncated ΔIS*CR2* downstream. The IV-type pattern of IS*CR2-tet*(X4)-ΔIS*CR2* was identified in 32 isolates, and its size was approximately 4.8 kb in length. These results indicated that IS*CR2* was the most common transposable element associated with *tet*(X4).

## 4. Discussion

In this study, the prevalence of *tet*(X4)-producing *E. coli* was mainly observed in Asia, especially in China (*n* = 465, 75.9%). Furthermore, the international spread of *tet*(X4)-producing *E. coli* isolates also mainly occurred in Asian countries, especially in China, Pakistan, Singapore, and Malaysia. The *tet*(X4) gene was first reported in China in 2019 [3,4]. The *tet*(X4) gene was first reported in Pakistan in 2021, which was detected in *E. coli* isolates from poultry, chicken meat, wild bird, and slaughterhouse wastewater [9]. In a retrospective survey of animal-derived tigecycline-resistant *E. coli* across China in 2018, the *tet*(X4) gene was not present at a high prevalence across China but was highly endemic in northwestern China [8]. It was noted that *tet*(X4)-producing *E. coli* isolates had emerged in several European countries, such as Italy, Norway, and Switzerland, where the genetic relationships of which were close to those from Asia. It is urgent to prevent the international spread of *tet*(X4)-producing *E. coli* isolates through timely intervention strategies.

Pigs have been found to be important vehicles of *tet*(X4)-producing *E. coli* isolates, which is consistent with the results from previous studies [4,5,8,16]. Furthermore, the pig *tet*(X4)-producing *E. coli* isolates from Shaanxi differed by only 119 SNPs from the human isolates in a previous study from China [8]. It was found in this study that the high genetic similarity of *tet*(X4)-producing *E. coli* isolates from animals (pigs and chickens) and humans (stools and bloods) was also observed, which suggested the possibility of its spread from animals to humans.

Importantly, *E. coli* isolates co-harboring *tet*(X4) and *mcr-1* have been reported [4,17,18]. In addition to *mcr-1*, carbapenems-resistant genes *bla*_NDM-5_ and *bla*_NDM-1_ were also identified in *tet*(X4)-producing *E. coli* isolates in this study. Furthermore, a gene cluster of *tmex*C-*tmex*D-*topr*J1 was identified in 7 isolates, which was a newly plasmid-mediated RND-type tigecycline resistance mechanism widespread among *Klebsiella Pneumoniae* isolates from food animals [19]. This co-existence of *tet*(X4) and other antibiotic resistance genes such as *mcr-1*, *bla*_NDM,_ and *tmex*C-*tmex*D-*topr*J1 would increase greatly the challenge for the treatment of pathogen infections, and this co-existence phenomenon might result from the co-selected pressure of extensively used antibiotics in animals and humans.

In previous studies, the IncX1-type plasmid was a common vector for *tet*(X4), which has a strong transmission ability and a wide host range [6,8]. It was interesting that no IncX1 replicon gene was found in this study, but that the IncX4 (4.5%) and IncX3 (1.8%) plasmid replicon genes were identified. In addition, multiple plasmid replicon genes were common in *tet*(X4)-producing *E. coli* isolates in this study. Indeed, *tet*(X4) has been reported in multi-replicon plasmids, such as IncX1-IncFIA/B-IncY, IncX-IncFIA/B-IncHI1A/B, IncFIA/B-IncHI1A/B, IncX1-IncN, and IncX1-IncR [8]. The combination of multiple plasmid replicons might contribute to their adaptation to a broad range of hosts through preventing plasmid incompatibility.

In this study, I-type (IS*26-tet*(X4)-IS*CR2*), II-type (ΔIS*1R-tet*(X4)-IS*CR2*), III-type (IS*CR2-tet*(X4)-IS*CR2*), and IV-type (IS*CR2-tet*(X4)-ΔIS*CR2*) transposon units were identified, which indicated that IS*CR2* was the most common mobile genetic element associated with *tet*(X4). IS*CR* elements are assumed to move and pick up adjacent sequences by rolling circle replication [20]. Therefore, IS*CR2* might play a key role in the transfer of *tet*(X4) among multi-replicon plasmids through transposition.

This study features some limitations. First, the number of genomes (*n* = 585) used in this study was limited, although they represent the full genome containing *tet*(X4) dataset in NCBI. Second, numerous genomes were associated with the development of sequencing technology. Third, the majority of the *tet*(X4)-positive *E. coli* isolates originated from China, and limited data were gathered from other countries. Therefore, the above limitations would bias the results found here.

## 5. Conclusions

In conclusion, *tet*(X4)-producing *E. coli* isolates have emerged in Asia and Europe. The international spread of these isolates can be primarily attributed to Asian countries, especially China, Pakistan, Singapore, and Malaysia. Pigs and their products are the most common sample vehicle of *tet*(X4)-producing *E. coli*. The mobile genetic element IS*CR2* might contribute to the spread of *tet*(X4). The spread of *tet*(X4) is a great concern for food safety and public health, and it is urgent to enhance surveillance and control its spread among *Enterobacteriaceae*.

## Data Availability

The data of this study are available from the authors upon reasonable request.

## Supplementary Materials

The following supporting information can be downloaded at: https://www.mdpi.com/article/10.3390/foods11142010/s1, Figure S1: Phylogenetic subtree of clade IV isolates, extracted from the tree in Figure 5; Table S1: AMR genotypes in *tet*(X4)-producing *E. coli* isolates.

## References

[1] Gerson S., Nowak J., Zander E., Ertel J., Wen Y., Krut O., Seifert H., Higgins P.G. (2018). Diversity of Mutations in Regulatory Genes of Resistance-Nodulation-Cell Division Efflux Pumps in Association with Tigecycline Resistance in Acinetobacter Baumannii. J. Antimicrob. Chemother..

[2] He F., Shi Q., Fu Y., Xu J., Yu Y., Du X. (2018). Tigecycline Resistance Caused by RpsJ Evolution in a 59-Year-Old Male Patient Infected with KPC-Producing *Klebsiella pneumoniae* during Tigecycline Treatment. Infect. Genet. Evol..

[3] He T., Wang R., Liu D., Walsh T.R., Zhang R., Lv Y., Ke Y., Ji Q., Wei R., Liu Z. (2019). Emergence of Plasmid-Mediated High-Level Tigecycline Resistance Genes in Animals and Humans. Nat. Microbiol..

[4] Sun J., Chen C., Cui C.-Y., Zhang Y., Liu X., Cui Z.-H., Ma X.-Y., Feng Y., Fang L.-X., Lian X.-L. (2019). Plasmid-Encoded *tet*(X) Genes That Confer High-Level Tigecycline Resistance in *E. coli*. Nat. Microbiol..

[5] Li Y., Wang Q., Peng K., Liu Y., Xiao X., Mohsin M., Li R., Wang Z. (2021). Distribution and Genomic Characterization of Tigecycline-Resistant *tet*(X4)-Positive *E. coli* of Swine Farm Origin. Microb. Genom..

[6] Cui C.-Y., Li X.-J., Chen C., Wu X.-T., He Q., Jia Q.-L., Zhang X.-J., Lin Z.-Y., Li C., Fang L.-X. (2022). Comprehensive Analysis of Plasmid-Mediated *tet*(X4)-Positive *E. coli* Isolates from Clinical Settings Revealed a High Correlation with Animals and Environments-Derived Strains. Sci. Total Environ..

[7] Bai L., Du P., Du Y., Sun H., Zhang P., Wan Y., Lin Q., Fanning S., Cui S., Wu Y. (2019). Detection of Plasmid-Mediated Tigecycline-Resistant Gene *tet*(X4) in *E. coli* from Pork, Sichuan and Shandong Provinces, China, February 2019. Eurosurveillance.

[8] Sun C., Cui M., Zhang S., Liu D., Fu B., Li Z., Bai R., Wang Y., Wang H., Song L. (2020). Genomic Epidemiology of Animal-Derived Tigecycline-Resistant *E. coli* across China Reveals Recent Endemic Plasmid-Encoded *tet*(X4) Gene. Commun. Biol..

[9] Mohsin M., Hassan B., Martins W.M.B.S., Li R., Abdullah S., Sands K., Walsh T.R. (2021). Emergence of Plasmid-Mediated Tigecycline Resistance *tet*(X4) Gene in *E. coli* Isolated from Poultry, Food and the Environment in South Asia. Sci. Total Environ..

[10] Bortolaia V., Kaas R.S., Ruppe E., Roberts M.C., Schwarz S., Cattoir V., Philippon A., Allesoe R.L., Rebelo A.R., Florensa A.F. (2020). ResFinder 4.0 for Predictions of Phenotypes from Genotypes. J. Antimicrob. Chemother..

[11] Liu B., Zheng D., Jin Q., Chen L., Yang J. (2019). VFDB 2019: A Comparative Pathogenomic Platform with an Interactive Web Interface. Nucleic Acids Res..

[12] Carattoli A., Zankari E., Garcia-Fernandez A., Larsen M.V., Lund O., Villa L., Aarestrup F.M., Hasman H. (2014). In Silico Detection and Typing of Plasmids Using PlasmidFinder and Plasmid Multilocus Sequence Typing. Antimicrob. Agents Chemother..

[13] Croucher N.J., Page A.J., Connor T.R., Delaney A.J., Keane J.A., Bentley S.D., Parkhill J., Harris S.R. (2015). Rapid Phylogenetic Analysis of Large Samples of Recombinant Bacterial Whole Genome Sequences Using Gubbins. Nucleic Acids Res..

[14] Stamatakis A. (2014). RAxML Version 8: A Tool for Phylogenetic Analysis and Post-Analysis of Large Phylogenies. Bioinformatics.

[15] Letunic I., Bork P. (2019). Interactive Tree Of Life (ITOL) v4: Recent Updates and New Developments. Nucleic Acids Res..

[16] Wang J., Wu H., Mei C.-Y., Wang Y., Wang Z.-Y., Lu M.-J., Pan Z.-M., Jiao X. (2021). Multiple Mechanisms of Tigecycline Resistance in *Enterobacteriaceae* from a Pig Farm, China. Microbiol. Spectr..

[17] Lu X., Xiao X., Liu Y., Li R., Wang Z. (2021). Emerging Opportunity and Destiny of *mcr*-1- and *tet*(X4)-Coharboring Plasmids in *E. coli*. Microbiol. Spectr..

[18] Sun H., Zhai W., Fu Y., Li R., Du P., Bai L. (2021). Co-Occurrence of Plasmid-Mediated Resistance Genes *tet*(X4) and *bla*_NDM-5_ in a Multidrug-Resistant *E. coli* Isolate Recovered from Chicken in China. J. Glob. Antimicrob. Res..

[19] Lv L., Wan M., Wang C., Gao X., Yang Q., Partridge S.R., Wang Y., Zong Z., Doi Y., Shen J. (2020). Emergence of a Plasmid-Encoded Resistance-Nodulation-Division Efflux Pump Conferring Resistance to Multiple Drugs, Including Tigecycline, in *Klebsiella pneumoniae*. mBio.

[20] Partridge S.R., Kwong S.M., Firth N., Jensen S.O. (2018). Mobile Genetic Elements Associated with Antimicrobial Resistance. Clin. Microbiol. Rev..

