# Supplementary material for: International Spread of Tet(X4)-Producing Escherichia coli Isolates
Source: Foods. 2022 Jul 7;11(14):2010. doi: 10.3390/foods11142010 (PMC9320811; doi:10.3390/foods11142010)
Supplement: Supplementary file 1 [file foods-11-02010-s001.zip › foods-1770224-supplementary.pdf]

foods

Communication

International Spread of Tet(X4)-Producing *Escherichia coli* Isolates

Zengfeng Zhang, Zeqiang Zhan and Chunlei Shi *

MOST-USDA Joint Research Center for Food Safety, School of Agriculture and Biology, and State Key Laboratory of Microbial Metabolism, Shanghai Jiao Tong University, Shanghai 200240, China; zhanzengfeng118@163.com (Z.Z.); zhanzeqiang@sjtu.edu.cn (Z.Z.)
* Correspondence: clshi@sjtu.edu.cn

Table S1. AMR genotypes in *tet*(X4)-producing *Escherichia coli* isolates.

Antibiotic resistance determinants		Numbers	Ratio/%
Carbapenems	*bla*$_{NDM-5}$	8	1.3
	bla$_{NDM-1}$	1	0.2
Colistin	*mcr*-1.1	77	12.6
	mcr-3.1	3	0.5
Tigecycline	*tmex*C-*tmex*D-*toprJ*1	7	1.1
Aminoglycosides	*dfr*A12	325	53.0
	*dfr*A14	110	17.9
	*dfr*A17	66	10.8
	*dfr*A5	52	8.5
	*dfr*A1	17	2.8
	aph(6)-Id	314	51.2
	aph(3'')-Ib	288	47.0
	aph(3')-Ia	173	28.2
	aph(4)-Ia	65	10.6
	*aad*A2	331	54.0
	*aad*A1	292	47.6
	*aad*A22	92	15.0
	*aad*A5	45	7.3
	aac(6')-IIc	1	0.2
	aac(6')-Ib-cr5	22	3.6
	aac(3)-IId	108	17.6
	aac(3)-IVa	65	10.6
	aac(3)-IIe	5	0.8
	aac(3)-IIg	1	0.2
β-Lactamase	*bla*$_{CTX-M-14}$	50	8.2
	bla$_{CTX-M-55}$	49	8.0
	bla$_{CTX-M-65}$	44	7.2
	bla$_{CTX-M-15}$	4	0.7
	bla$_{CTX-M-3}$	3	0.5
	bla$_{CTX-M-24}$	3	0.5
	bla$_{CMY-2}$	18	2.9
	bla$_{TEM}$	545	88.9
	bla$_{SHV-12}$	51	8.3
	bla$_{DHA-1}$	4	0.7
	bla$_{OXA-1}$	12	2.0
	bla$_{OXA-10}$	14	2.3
	bla$_{OXA-232}$	3	0.5
Rifampicin	*arr-3*	18	2.9
Chloramphenicol	*cml*A1	239	39.0
	*cat*B3	22	3.6

	*cat*A2	14	2.3
Macrolides	*mph*(A)	168	27.4
	mef(B)	118	19.2
	erm(B)	70	11.4
	erm(42)	59	9.6
Fosfomycin	*fos*A3	49	8.0
	*fos*A4	35	5.7
Florfenicol	*flo*R	584	95.3
Lincomycin	*lnu*(F)	142	23.2
	lnu(G)	148	24.1
Sulfonamide	*sul*3	417	68.0
	*sul*2	340	55.5
	*sul*1	101	16.5
Tetracyclines	*tet*(A)	501	81.7
	tet(M)	226	36.9
	tet(B)	127	20.7
Quinolones	*qnr*S1	381	62.2
	*oqx*A	42	6.9
	*oqx*B	42	6.9
	*qnr*S2	19	3.1
	*qnr*B4	4	0.7
	*qep*A1	3	0.5
	*qnr*B19	2	0.3
	*qnr*B2	1	0.2
AMR Mutations	*glp*T_E448K	351	57.3
	*gyr*A_S83L	242	39.5
	*par*C_S80I	163	26.6
	*gyr*A_D87N	139	22.7
	*uhp*T_E350Q	46	7.5
	*par*E_S458A	37	6.0
	*cya*A_S352T	31	5.1
	*par*C_A56T	20	3.3
	*nfs*A_G131D	14	2.3
	*sox*S_A12S	9	1.5
	*nfs*A_R15C	7	1.1
	*par*E_L416F	4	0.7
	*par*E_I355T	2	0.3
	*nfs*A_Q44STOP	2	0.3
	*mar*R_S3N	1	0.2

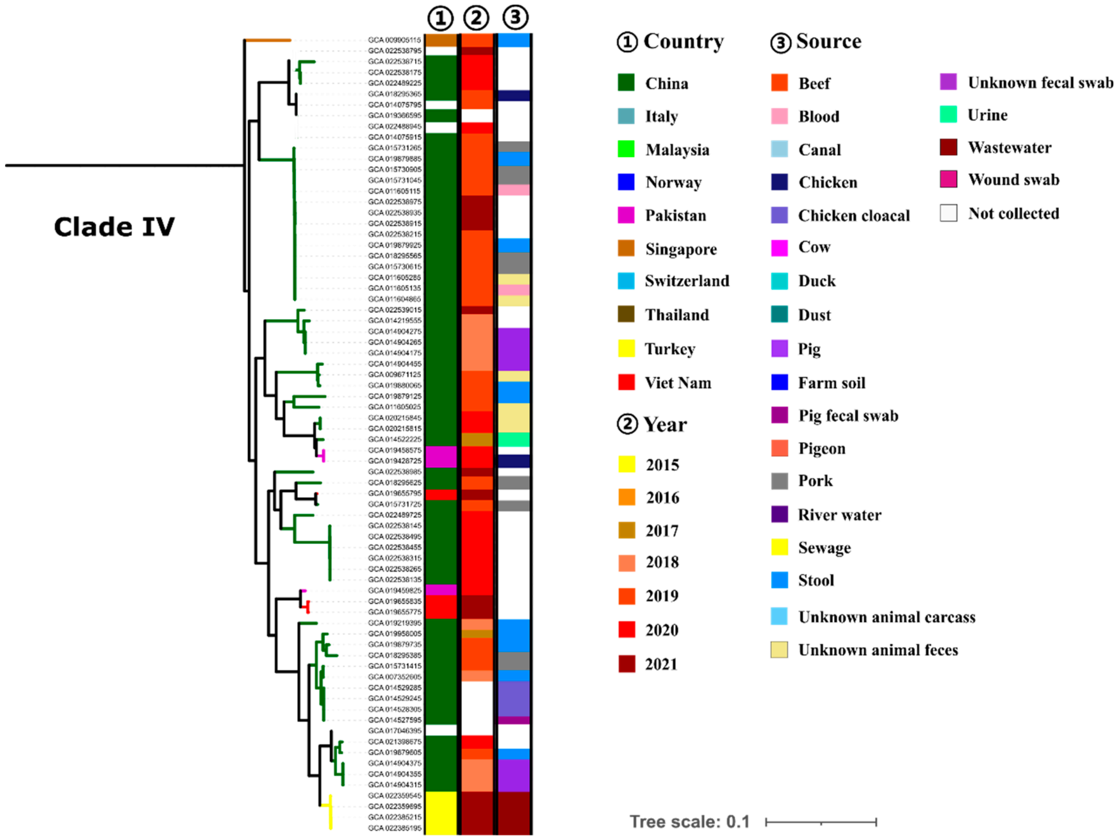

Figure S1. Phylogenetic subtree of Lineage IV isolates extracted from the tree in Figure 5. Column ① depicts isolate isolation countries; ② denotes the distribution of years; ③ denotes sample sources. The detailed information in ①-③ is shown in the key using various colors.
